# Nephrotic syndrome sera induce different transcriptomes in podocytes based on the steroid response

**DOI:** 10.14814/phy2.15932

**Published:** 2024-02-02

**Authors:** Martin Bezdicka, Ondrej Cinek, Valerij Semjonov, Katerina Polackova, Eva Sladkova, Jakub Zieg, Moin A. Saleem, Ondrej Soucek

**Affiliations:** ^1^ Vera Vavrova Lab/VIAL, Department of Pediatrics, Second Faculty of Medicine Charles University and Motol University Hospital Prague Czech Republic; ^2^ Department of Pediatrics, Second Faculty of Medicine Charles University and Motol University Hospital Prague Czech Republic; ^3^ Children's Clinic, Faculty of Medicine in Pilsen University Hospital in Pilsen, Charles University Pilsen Czech Republic; ^4^ Bristol Renal and Bristol Royal Hospital for Children University of Bristol Medical School Bristol UK

**Keywords:** human immortalized podocytes, nephrotic syndrome, RNA sequencing, serum, transcriptome

## Abstract

As the molecular mechanism of nephrotic syndrome remains largely undiscovered, patients continue to be exposed to the pros and cons of uniform glucocorticoid treatment. We explored whether the exposure of in vitro‐cultivated podocytes to sera from children with steroid‐sensitive or steroid‐resistant nephrotic syndrome induces differences in gene expression profiles, which could help to elucidate the pathogenesis of the steroid response. Human immortalized podocytes were cultivated with patient sera for 3 days. After cell lysis, RNA extraction, 3′‐mRNA libraries were prepared and sequenced. There were 34 significantly upregulated and 14 downregulated genes (fold difference <0.5 and >2.0, respectively, and false discovery rate‐corrected *p* < 0.05) and 22 significantly upregulated and 6 downregulated pathways (false discovery rate‐corrected *p* < 0.01) in the steroid‐sensitive (*n* = 9) versus steroid‐resistant group (*n* = 4). The observed pathways included upregulated redox reactions, DNA repair, mitosis, protein translation and downregulated cholesterol biosynthesis. Sera from children with nephrotic syndrome induce disease subtype‐specific transcriptome changes in human podocytes in vitro. However, further exploration of a larger cohort is needed to verify whether clinically distinct types of nephrotic syndrome or disease activity may be differentiated by specific transcriptomic profiles and whether this information may help to elucidate the pathogenesis of the steroid response.

## Graphical abstract

Visualization of methods, outcome and conclusion of the nephrotic syndrome serum study.

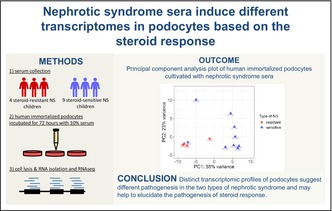

## 1 INTRODUCTION

Nephrotic syndrome (NS) is characterized by nephrotic proteinuria, hypoalbuminemia, edema, and hyperlipidemia and occurs on average 2.92 (range 1.15–16.9) per 100,000 children per year (Veltkamp et al., 2021). Approximately 85%–95% of children with NS respond to the initial 4–6 week high‐dose glucocorticoid treatment by disappearance of the symptoms and are thus classified as steroid‐sensitive NS (Sinha & Bagga, 2022), although most of them relapse (Rovin et al., 2021; Trautmann et al., 2023). The remaining 5%–15% of children do not achieve remission and are thus termed steroid‐resistant NS (Sinha & Bagga, 2022). The etiology of NS is largely unknown: only a fraction of cases can be explained by causative gene variants, all of which are classified as steroid resistant.

Moreover, nephrotic syndrome may be caused by a circulating factor(s). It has been shown that injection of human NS plasma causes proteinuria in healthy rats (Sharma et al., 2002) and that transient NS may occur in neonate of a mother with NS (Kemper et al., 2001). To date, immunological serum circulating factors are known to target podocytes and affect their structure and function (Saleem, 2019). Examples of serum factor candidates include hemopexin (Lennon et al., 2008), cardiotrophin‐like cytokine factor 1 (Savin et al., 2015), soluble urokinase receptor, CD40 autoantibodies, CASK (Candelier & Lorenzo, 2020), and nephronectin (Watany & El‐Horany, 2018). Most of these factors were associated with either of the typical NS histological types (minimal change disease [MCD] or focal segmental glomerulosclerosis [FSGS]). Recently, published proteomic and cytokine profiling studies suggested protein panels that could help to predict steroid resistance prior to glucocorticoid treatment (Agrawal et al., 2020, 2021). In our study, we used human immortalized podocytes, a widely used and verified cellular model of differentiated human podocytes (Ni et al., 2012), to test whether exposure of these in vitro cultivated human podocytes to sera from patients with steroid‐sensitive versus steroid‐resistant NS induces distinct gene expression profiles.

## 2 MATERIALS AND METHODS

### 2.1 Ethics statement

The study protocol was approved by the Motol University Hospital Ethics Committee, and we confirm that all methods were carried out in accordance with relevant guidelines and regulations and the Code of Ethics of the World Medical Association (Declaration of Helsinki). The study subjects and/or their guardians provided written informed consent with the study and with the EU GDPR principles of data protection. The data were pseudonymized before the analysis.

### 2.2 Serum samples

The present study used sera from 13 children with NS—nine with steroid‐sensitive NS and four with steroid‐resistant NS. The nine steroid‐sensitive NS serum samples originated from biobanked samples taken upon diagnosis from treatment‐naive individuals (between October 2018 and April 2022) who subsequently responded to the initial 6‐week course of glucocorticoid treatment by remission of the proteinuria. Steroid‐resistant NS samples were obtained from patients with known steroid‐resistant NS regularly followed up at the Prague Centre, who experienced long‐term disease remission and at least 6 months after cyclosporine discontinuation at the time of blood draw. All steroid‐resistant NS patients were treated initially with glucocorticoids and after the confirmation of the steroid resistance with cyclosporine and ACE inhibitors. In addition to patient CZ5837, children with steroid‐resistant NS were also treated with ACE inhibitors at the time of blood draw. Blood samples were centrifuged approximately 60 min after collection, and the serum was carefully separated, frozen, and stored at −80°C. All clinical data are available in Table 1.

**TABLE 1:** Clinical data of patients with nephrotic syndrome.

Type of NS	Steroid‐sensitive										Steroid‐resistant					Unpaired t test steroid‐sensitive vs. resistant NS (*p* value)
ID	CZ12212	CZ11915	CZ12655	CZ12983	CZ13508	CZ13702	CZ14087	CZTRN01	CZ14054	Mean ± SD	CZ5837	CZ9019	CZ9488	CZ11136	Mean ± SD	
Sex	M	M	F	M	F	M	F	F	M		F	F	M	M		
Age at disease onset (years)	6.0	3.5	5.0	5.0	4.0	2.0	4.5	2.8	2.0	3.9 ± 1.4	2.7	5.7	10.7	6.2	6.3 ± 3.3	0.247
Weight (kg)	26.0	23.0	19.4	18.5	20.3	16.0	21.0	16.8	16.0	19.7 ± 3.4	14.4	20.9	75.7	26.4	34.4 ± 28	0.373
Height (cm)	123	107	105	114	106	93	112	102	87	105.4 ± 10.8	90	114	154	124	120.5 ± 26.5	0.353
Height Z‐score	1.3	2.2	−0.6	1.1	1.2	1.8	1.7	2.7	0.1	1.4 ± 0.8	−0.8	0.1	1.6	1.4	1.0 ± 0.7	0.349
BMI Z‐score	1.1	2.8	1.4	−1.2	1.6	1.2	1.1	0.2	2.4	1.4 ± 0.8	1.2	0.7	2.5	1.1	1.4 ± 0.8	0.887
Edemas	Yes	Yes	Yes	No	Yes	Yes	Yes	Yes	Yes	‐	Yes	Yes	No	No	‐	‐
Hypertension	Yes	Yes	No	No	Yes	Yes	No	Yes	Yes	‐	Yes	No	No	No	‐	‐
Hematuria	No	No	No	Yes	No	Yes	No	No	Yes	‐	No	No	No	No	‐	‐
Extrarenal complications	No	No	SPE	No	No	No	No	SCR	No	‐	No	No	No	No	‐	‐
Serum‐creatinine (μmol/L)	31	15	36	41	35	46	23	23	24	30.4 ± 10	28	27	42	27	31 ± 7.4	0.914
Serum total protein (g/L)	44	35	40	39	55	40	36	40	43	41.3 ± 5.9	52	64	62	52	57.5 ± 6.4	0.008
Serum albumin (g/L)	24	15	14	14	19	19	20	25	18	18.7 ± 4	34	43	38	33	37.0 ± 4.6	0.0009
eGFR (mL/min/1.73 m^2^)	145	260	108	102	145	60	>150	>150	130		117	>150	134	>150		
Urine protein/creatinine (mg/mmol)	506	889	339	1445	1026	420	1863	1698	1770	1106.2 ± 607.4	11	38	15	15	19.8 ± 12.3	0.0007
Renal biopsy finding	ND	ND	MCD	None	ND	ND	ND	ND	ND	‐	FSGS	MCD	FSGS	MCD	‐	‐
Time since the end of immunosuppression in steroid‐resistant NS (years)	‐										9	2.3	1.1	0.5	3.2 ± 3.4	‐

*Note*: The clinical data and serum samples were obtained upon manifestation of steroid‐sensitive group and at regular follow‐up and serum sampling of patients with steroid‐resistant NS.

Abbreviations: FSGS, focal segmental glomerulosclerosis; MCD, minimal change disease; ND, not done; SCR, suspicion of cardiac rhabdomyoma; SPE, small pericardial effusion.

### 2.3 Cultivation of podocytes

Human podocytes, which were originally derived from 3‐year‐old female donor with unilateral obstructive/reflux nephropathy (and no primary glomerular disease) (Saleem et al., 2002), were immortalized with the thermosensitive *SV40‐T* gene and were provided by Prof. Moin A Saleem (University of Bristol, United Kingdom). The cells were cultured at 33°C and 5% CO_2_ in RPMI 1640 medium (Thermo Fisher Scientific, 21875034) supplemented with 10% heat‐inactivated fetal bovine serum (Sigma–Aldrich, F9665), 1% insulin‐transferrin‐selenium (Thermo Fisher Scientific, 41400045), and 1% penicillin–streptomycin (Thermo Fisher Scientific, 15140122). After reaching 80% confluence, the cells were split and seeded into 60 mm Petri dishes at a density of 100,000 cells/dish. According to the standard protocol (Saleem et al., 2002), the shift from proliferation to differentiation (by changing the cultivation temperature from 33°C to 37°C) was performed 72 h later (concurrently with removing penicillin–streptomycin from the cultivation medium). After 14 days, fully differentiated cells were cultivated with 10% human serum in triplicate for each patient. Seventy‐two hours later, the cells were lysed, and RNA was isolated with TRIzol and PureLink RNA Mini kits according to the manufacturer's protocol (Thermo Fisher Scientific, 12183555). The RNA was aliquoted and stored at −80°C. The 10% serum concentration was selected based on our preexperimental protocol optimization, which yielded significant differences in gene expression with no signs of cell death or shape abnormalities after more than 3 days of cultivation. Whereas a lower serum concentration (7.5%) resulted in no cellular response, higher concentrations (15% and 30%) were toxic.

### 2.4 RNA sequencing and data analysis

Three technical podocyte culture replicates were planned for each serum sample. After incubation, the cells were lysed, and their RNA was extracted and sequenced independently. The RNA quality was measured by an Agilent 2100 Bioanalyzer (Agilent RNA Nano Kit 5067‐1511 by Agilent Technologies, Santa Clara, CA). The quality RIN was between 8 and 10 for all RNA samples. For a single serum sample, only two technical replicates were obtained due to unexpected cell death in one of the three Petri dishes.

The 3′‐mRNA sequencing libraries were prepared using the Collibri 3′ mRNA Library Prep Kit (cat. no A38110096; Thermo Fisher Scientific, Waltham, MA) and sequenced unidirectionally with a read length of 163 bases on a NextSeq instrument using the NextSeq 500/550 High Output Kit v2.5 (150 cycles) (both Illumina, Foster City, CA). All replicates were sequenced in one run. The resulting demultiplexed fastq files were downloaded from BaseSpace, and adapters were trimmed using Cutadapt (Martin, 2011). Alignments against the human genome (hg38) were performed with the Gencode annotation release 38 (May 2021), along with gene counting by the STAR aligner (Dobin et al., 2013), with parameters mostly set as advised by the manufacturer of the Collibri library construction kit.

**FIGURE 1:**
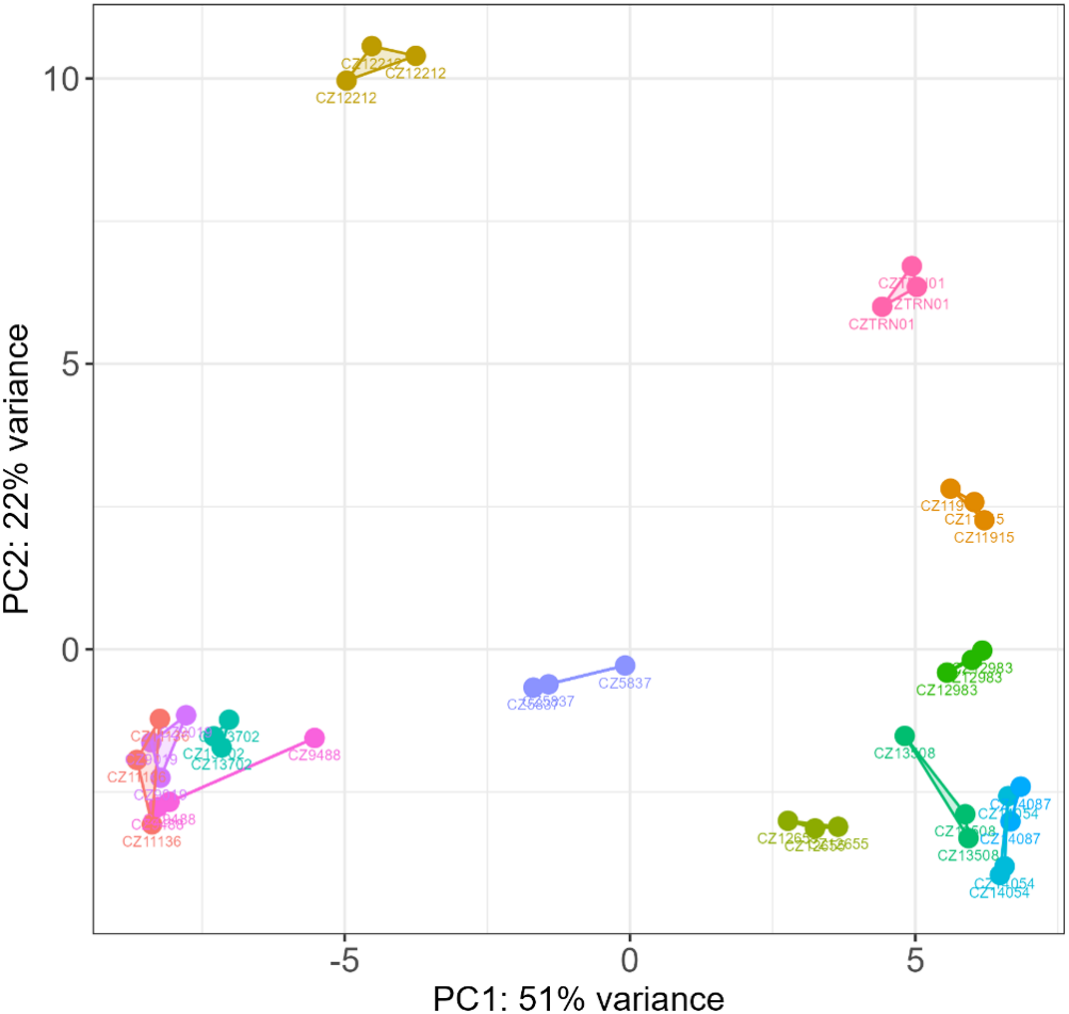
Principal component analysis of transcriptomic profiles of podocytes cultivated with 10% serum samples from children with nephrotic syndrome—before merging technical replicates.

The gene count tables and the sample metadata were assembled into a DESeq2 *DESeqDataSet* objective (Love et al., 2014), and the mutual distances of the technical replicates were inspected via a principal component analysis (PCA) plot (Figure 1). After verifying their similarity, technical replicates were merged, which resulted in 14.9–28.1 million reads per sample.

**FIGURE 2:**
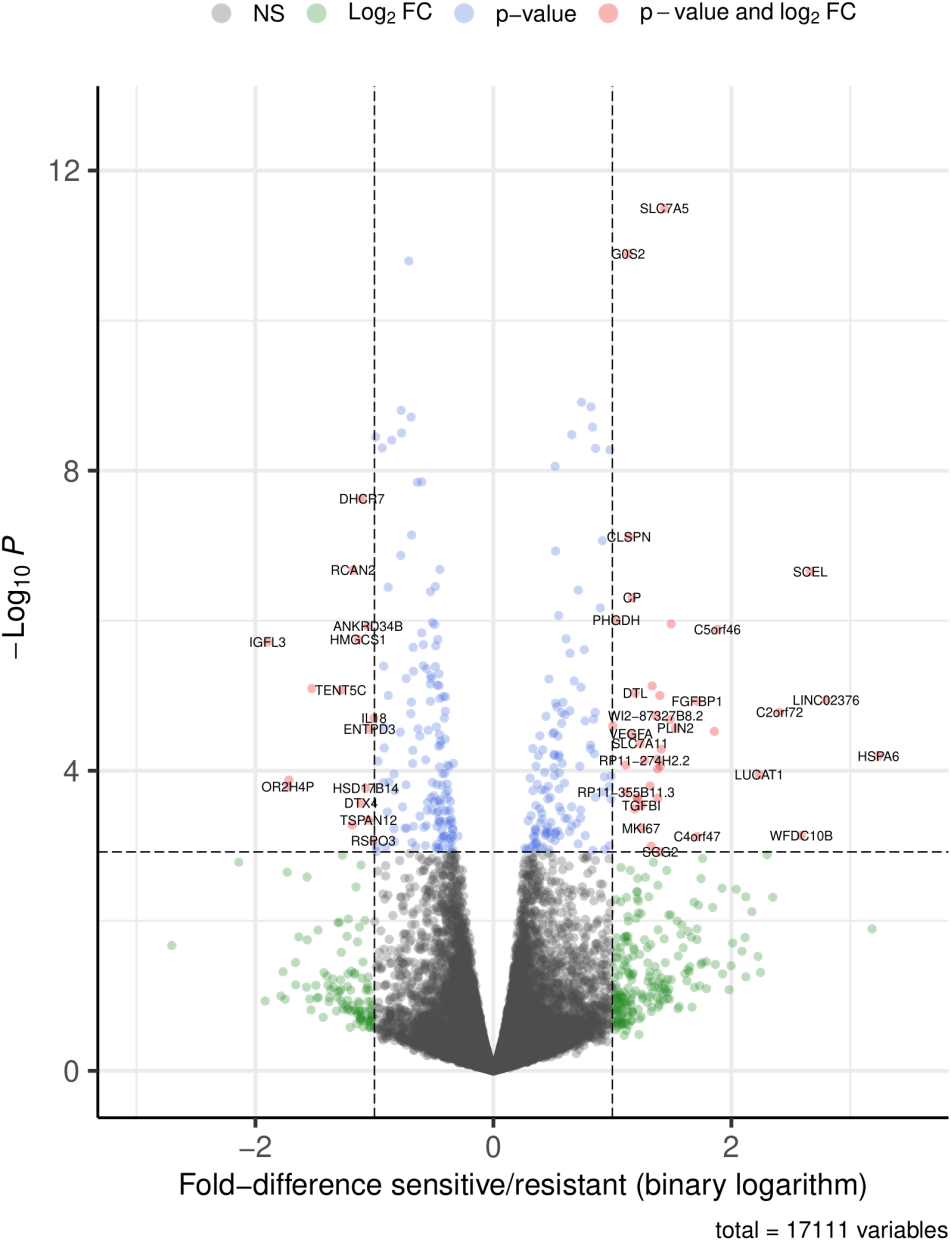
Volcano plot illustrating gene expression differences between steroid‐sensitive and steroid‐resistant NS serum samples in podocytes. FC, fold change; NS, non‐significant. Nine genes (*IGFL3, OR2H4P, RP11‐93B14.6, C4orf47, FGFBP1, C2orf72, WFDC10B, SCEL, and HSPA6*) showed here were excluded by DESeq2 automatic independent filtering function and are thus not included in Table 2.

Genes were filtered for having 10 reads or more in at least three different samples; 17241 genes were retained. Global differences in gene expression between steroid‐sensitive tissue culture‐derived serum and steroid‐resistant serum from NS patients (conditions sensitive vs. resistant) were assessed via ordination plots, and particular DEGs were identified via a negative binomial regression model in DESeq2. The results were visualized using MA plots, volcano plots (Figure 2), *p* value distribution plots, heatmaps and count plots of the genes of interest. The DESeq test *P* values were corrected for multiple testing using the Benjamini–Hochberg method. A minimum of twofold increase or decrease in expression and a corrected *p* value <0.05 were needed for a gene to be considered differentially expressed. Gene count tables and raw sequencing data were deposited at the NCBI Gene Expression Omnibus (GEO) under accession code GSE215231.

The web‐based application Reactome (https://reactome.org, version 84) was used to perform pathway enrichment analysis. Camera was selected as the analysis method (correlation adjusted mean rank), which is based on a two‐sided *t* test allowing for correlation (Wu & Smyth, 2012). The test statistic is modified by the variance inflation factor estimated from the data. Hence, the intergene correlation was taken into account. We tested whether the fold differences resulting from the application of serum differed between the gene set of interest and the background. Steroid‐resistant NSs were used as a reference group. The FDR approach was applied to adjust for multiple testing procedures. The threshold for the FDR‐adjusted *p* value (*Q* value) was set to 0.01. Even slight intergene correlations can seriously increase the FDR. The principal advantage of Camera over widely used GSEA methods is its ability to adjust for intergene correlations and control FDRs correctly. In addition, compared to GSEA and other methods, Camera does not depend on sample permutation, which is particularly useful when the number of samples is small. Hence, an undesirable decrease in statistical power can be avoided when using Camera instead of GSEA.

## 3 RESULTS

**FIGURE 3:**
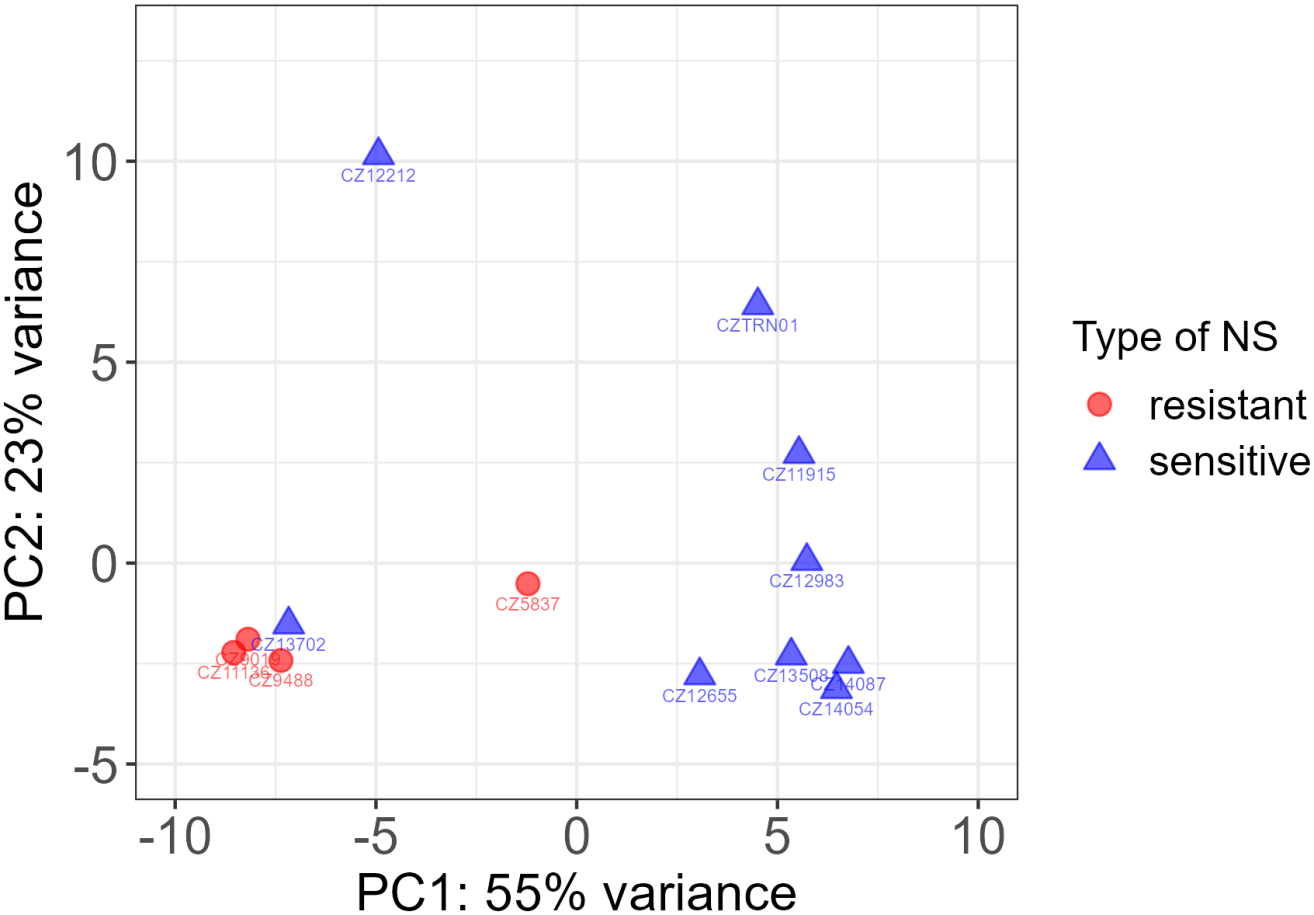
Principal component analysis of transcriptomic profiles of podocytes cultivated with 10% serum samples from children with nephrotic syndrome.

The serum‐induced transcriptome profiles of the cultured podocytes differed depending on whether the sera originated from steroid‐sensitive or steroid‐resistant NS patients (Figure 3). There were 48 significantly differentially expressed genes (fold difference >2.0 or <0.5 and false discovery rate‐corrected *p* < 0.05), 34 of which were upregulated and 14 of which were downregulated in cells exposed to steroid‐sensitive NS sera compared with those exposed to steroid‐resistant NS sera (Table 2). An ordination (PCA) revealed a tight cluster of steroid‐resistant NS serum‐induced transcriptome profiles. One presumably steroid‐sensitive NS profile clustered along with the steroid‐resistant profile. The steroid‐sensitive NS serum‐induced profile was spread more broadly but was well separated from the steroid‐resistant NS serum‐induced profile (Figure 3).

**TABLE 2:** Significantly differentially expressed genes in human immortalized podocytes cultured with steroid‐sensitive and steroid‐resistant NS serum samples.

Gene	Function (according to GeneCards)	*p*_adj_	Fold‐difference
*LINC02376*	Long Intergenic Non‐Protein Coding RNA	0.002	6.949
*LUCAT1*	Long Intergenic Non‐Protein Coding RNA	0.009	4.703
*C5orf46*	Unknown	<0.001	3.687
*LINC02274*	Long Intergenic Non‐Protein Coding RNA	0.003	3.621
*PLIN2*	Structural component of lipid droplets	0.003	2.886
*FAM111B*	Serine protease	<0.001	2.815
*DDIT4*	Regulator of cell growth, proliferation and survival through mTORC1	0.003	2.800
*SLC7A5*	High‐affinity transporter that mediates uptake of large neutral amino acids	<0.001	2.708
*ANGPTL4*	Regulator of glucose homeostasis, lipid metabolism, and insulin sensitivity	0.005	2.659
*DGCR5*	Long Intergenic Non‐Protein Coding RNA	0.007	2.646
*SCG2*	Regulator of the biogenesis of secretory granules	0.047	2.643
*PCLAF*	Regulator of DNA repair during DNA replication	0.002	2.637
*POU5F1*	Transcription factor in embryonic development and stem cell pluripotency	0.015	2.607
*RPL17P50*	Pseudogene	0.008	2.604
*LRAT*	Enzyme in vitamin A metabolism	0.006	2.602
*WI2‐87327B8.2*	Long Intergenic Non‐Protein Coding RNA	0.003	2.572
*NCKAP5*	Microtubule bundle formation and microtubule depolymerization	0.001	2.522
*SYTL3*	Vesicle trafficking	0.011	2.491
*RP11‐274H2.2*	Unknown	0.007	2.408
*TGFBI*	TGFB‐induced, cell adhesion modulator, cell‐collagen interactions	0.018	2.367
*MKI67*	Regulation of chromosome segregation and mitotic nuclear division	0.029	2.365
*SLC7A11*	Antiporter mediating the exchange of extracellular anionic L‐cystine and intracellular L‐glutamate across the cellular plasma membrane	0.005	2.349
*RP13‐516 M14.10*	Unknown	0.015	2.320
*PTGS1*	Enzyme in the conversion of arachidonate to prostaglandin	0.015	2.308
*DTL*	Regulator in protein ubiquitination and G2/M transition of mitotic cell cycle	0.002	2.284
*LINC00702*	Long Intergenic Non‐Protein Coding RNA	0.019	2.277
*CP*	Peroxidation of Fe(II)transferrin to Fe(III)transferrin	<0.001	2.242
*VEGFA*	Growth factor, regulation of IL4, IL13	0.004	2.228
*CLSPN*	Activator of checkpoint arrest of the cell cycle in response to replicative stress or DNA damage	<0.001	2.202
*G0S2*	Activator of extrinsic apoptotic signaling pathway	<0.001	2.193
*RP11‐355B11.3*	Unknown	0.013	2.165
*B4GALNT2*	Enzyme in biosynthesis of the human Sd(a) antigen	0.007	2.163
*PHGDH*	Enzyme in L‐serine biosynthesis	<0.001	2.045
*RP11‐177H13.2*	Unknown	0.003	2.003
*IL18*	Pro‐inflammatory cytokine of the IL‐1 family	0.003	0.499
*RSPO3*	Regulation of Wnt signaling pathway	0.037	0.496
*ENTPD3*	Regulation of nucleotide metabolism	0.003	0.486
*TSPAN12*	Role in receptor signal transduction	0.024	0.483
*ANKRD34B*	Cytosolic phosphoprotein	<0.001	0.482
*HSD17B14*	Dehydrogenase in steroid metabolism	0.012	0.475
*DHCR7*	Enzyme in cholesterol biosynthesis	<0.001	0.465
*DTX4*	Regulator of Notch signaling	0.017	0.462
*HMGCS1*	Enzyme in cholesterol biosynthesis	<0.001	0.455
*RCAN2*	Regulator of calcineurin A	<0.001	0.441
*SCNN1A*	Subunit of epithelial sodium channel	0.027	0.439
*TENT5C*	mRNA stabilization	0.001	0.411
*SLITRK4*	Synaptogenesis, synapse differentiation, regulation of neurite growth	0.001	0.347
*ALDH1A1*	Aldehyde dehydrogenase in alcohol metabolism	0.010	0.304

*Note*: *p*_adj_ = Benjamini–Hochberg adjusted *p*‐value.

Gene set enrichment analysis revealed 28 cellular pathways differentially regulated between the steroid‐sensitive and steroid‐resistant serum‐induced NS patient profiles (Table 3). Most of these pathways (22 out of 28) were upregulated in cells exposed to steroid‐sensitive NS sera and were involved in redox reactions (selenocysteine production), DNA repair mechanisms, mitosis, and protein synthesis (translation). The downregulated cellular processes in steroid‐sensitive NS serum‐induced podocytes (6 out of 28) all represented cholesterol biosynthesis.

**TABLE 3:** Significantly regulated pathways in human immortalized podocytes cultivated with steroid‐sensitive versus steroid‐resistant NS serum samples.

Cellular process	Pathway regulation	*Q* value	Pathway name and enrichment plot
Cell cycle checkpoints	Increased in steroid‐sensitive NS	0.002	Activation of ATR in response to replication stress 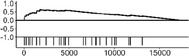
Cell cycle, mitotic	Increased in steroid‐sensitive NS	0.006	Resolution of Sister Chromatid Cohesion 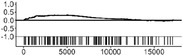
Cellular responses to stimuli	Increased in steroid‐sensitive NS	0.002	Response of EIF2AK4 (GCN2) to amino acid deficiency 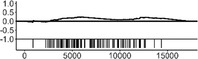
DNA repair	Increased in steroid‐sensitive NS	0.002	Homologous DNA Pairing and Strand Exchange 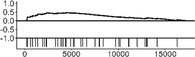
DNA repair	Increased in steroid‐sensitive NS	0.005	Presynaptic phase of homologous DNA pairing and strand Exchange 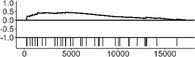
DNA repair	Increased in steroid‐sensitive NS	0.010	HDR through Homologous Recombination (HRR) 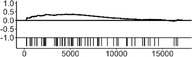
DNA repair	Increased in steroid‐sensitive NS	0.010	Resolution of D‐loop Structures through Synthesis‐Dependent Strand Annealing (SDSA) 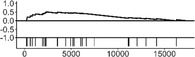
DNA Replication	Increased in steroid‐sensitive NS	0.002	DNA strand elongation 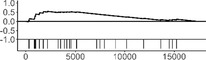
DNA Replication	Increased in steroid‐sensitive NS	0.002	Activation of the pre‐replicative complex 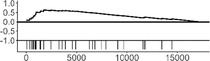
Metabolism of amino acids and derivatives	Increased in steroid‐sensitive NS	0.004	Selenocysteine synthesis 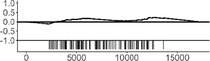
Metabolism of amino acids and derivatives	Increased in steroid‐sensitive NS	0.006	Selenoamino acid metabolism 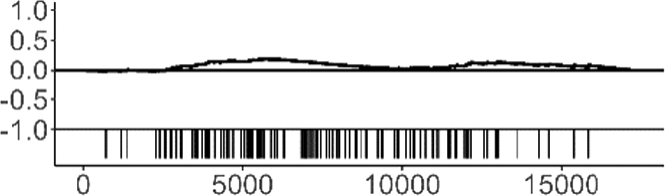
Metabolism of lipids	Decreased in steroid‐sensitive NS	<0.001	Cholesterol biosynthesis 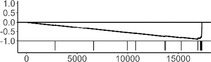
Metabolism of lipids	Decreased in steroid‐sensitive NS	<0.001	Activation of gene expression by SREBF (SREBP) 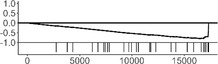
Metabolism of lipids	Decreased in steroid‐sensitive NS	<0.001	Cholesterol biosynthesis via desmosterol 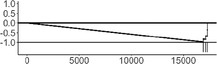
Metabolism of lipids	Decreased in steroid‐sensitive NS	<0.001	Cholesterol biosynthesis via lathosterol 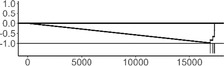
Metabolism of lipids	Decreased in steroid‐sensitive NS	<0.001	Metabolism of steroids 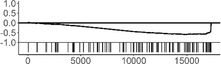
Metabolism of lipids	Decreased in steroid‐sensitive NS	0.001	Regulation of cholesterol biosynthesis by SREBP (SREBF) 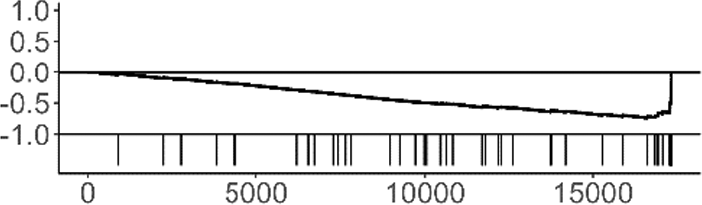
Metabolism of proteins	Increased in steroid‐sensitive NS	0.001	L13a‐mediated translational silencing of Ceruloplasmin expression 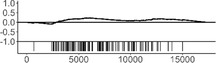
Metabolism of proteins	Increased in steroid‐sensitive NS	0.002	GTP hydrolysis and joining of the 60S ribosomal subunit 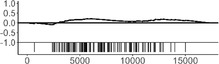
Metabolism of proteins	Increased in steroid‐sensitive NS	0.002	Eukaryotic Translation Elongation 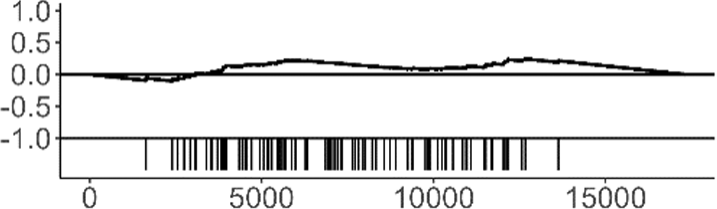
Metabolism of proteins	Increased in steroid‐sensitive NS	0.002	Eukaryotic Translation Initiation 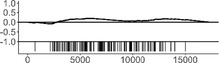
Metabolism of proteins	Increased in steroid‐sensitive NS	0.002	Cap‐dependent Translation Initiation 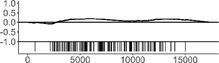
Metabolism of proteins	Increased in steroid‐sensitive NS	0.002	Peptide chain elongation 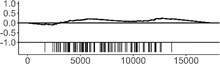
Metabolism of proteins	Increased in steroid‐sensitive NS	0.002	Formation of a pool of free 40S subunits 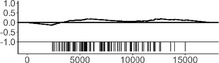
Metabolism of proteins	Increased in steroid‐sensitive NS	0.010	SRP‐dependent cotranslational protein targeting to membrane 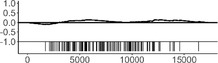
Metabolism of RNA	Increased in steroid‐sensitive NS	0.006	Nonsense‐Mediated Decay (NMD) 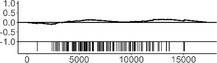
Metabolism of RNA	Increased in steroid‐sensitive NS	0.006	Nonsense Mediated Decay (NMD) enhanced by the Exon Junction Complex (EJC) 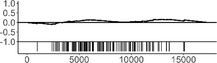
Metabolism of RNA	Increased in steroid‐sensitive NS	0.009	Nonsense Mediated Decay (NMD) independent of the Exon Junction Complex (EJC) 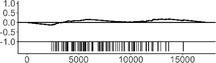

*Note*: The enrichment score was calculated according to GSEA (details in the Supplementary methods). The values on the *x*‐axis represent the decreasing order of genes ranked by the signal‐to‐noise ratio. The y‐axis represents the running enrichment score. The *Q* value represents the threshold for an FDR‐adjusted *p* value, which was set to 0.01 according to the Camera online software (Wu & Smyth, 2012).

## 4 DISCUSSION

Our results reveal differential reactions of a classic podocyte cell model to exposure to sera from treatment‐naive patients with steroid‐sensitive NS compared to those of a classic podocyte cell model exposed to sera from patients with long‐term remission of steroid‐resistant NS at least 6 months after discontinuation of immunosuppressive treatment. These findings suggest that clinically distinct subtypes of NS might have specific impacts on podocytes and thus distinct pathophysiologies.

An ordination by PCA identified a tight cluster formed by transcription profiles induced by three steroid‐resistant and one steroid‐sensitive NS serum sample. The reason for the clustering of steroid‐sensitive NS serum CZ13702 levels together with steroid‐resistant NS profiles is presently unknown; the patient's case was not clinically remarkable apart from his low glomerular filtration rate during the manifestation of the disease (Table 1). The clinical data of patient CZ13702 were not different from those of the other steroid‐sensitive NS patients. Moreover, as patient CZ13702 was sampled while having active NS and steroid‐resistant NS patients were sampled at disease remission, it is highly improbable that the clustering on the PCA plot was due to disease activity (rather than to glucocorticoid treatment response). Of the steroid‐resistant NS profiles, the outlier sample CZ5837 originated from a patient who manifested earlier (2.7 vs. 6.3 ± 2.9 years) and had much longer disease remission (9.0 vs. 3.2 ± 3.3 years) than the other steroid‐resistant NS patients (Table 1). As neither of our patients with steroid‐resistant NS carried causative variants in any of the 69 genes associated with NS (Bezdíčka et al., 2018), genetics could not explain this outlier.

The dispersion of transcriptome profiles induced by steroid‐sensitive NS sera was wider than the dispersion induced by steroid‐resistant NS sera. We speculate that this difference reflects the differential intensity of the putative serum‐mediated process in individual treatment‐naive patients upon diagnosis. The observed increase in spread somewhat resembles the greater variability noted in the transcriptome profiles of peripheral blood mononuclear cells from steroid‐sensitive NS patients than in those from steroid‐resistant patients (Kang et al., 2017).

Gene set enrichment analysis (GSEA) revealed several pathways differentially influenced by steroid‐sensitive vs. steroid‐resistant NS sera. We observed the upregulation of the synthesis of selenocysteine, an amino acid important in redox reactions (Rayman, 2012), and increased DNA repair, which suggested that certain stress in podocytes was induced by steroid‐sensitive NS sera. In addition, upregulated mitosis and protein translation highlight that podocytes actively react to steroid‐sensitive NS sera with the aim of adapting and surviving (Coward et al., 2005). The downregulated cholesterol biosynthesis may be just a physiological reaction to the increased cholesterol concentration in steroid‐sensitive NS serum samples, as podocytes possess a mechanism to protect themselves from the deleterious effects of hypercholesterolaemia (Vaziri, 2016).

This comparison is not straightforward because of the scarcity of available studies on the transcriptomics of NS. Kang et al. investigated the transcriptome profiles of peripheral blood mononuclear cells rather than cell cultures, testing differences between four steroid‐resistant and six steroid‐sensitive NS patients with active disease (Kang et al., 2017). Based on the overrepresentation analysis, 23 differentially expressed genes were found to be linked to TGF‐β signaling, glycerol phospholipid biosynthesis, redox reactions, and protein synthesis. Although none of the differentially expressed genes were found to be differentially expressed in our study, the other two cellular processes are involved; that is, both blood mononuclear cells and podocytes increase their redox and proteosynthetic capacity. Later, Panigrahi et al. investigated the gene expression of human immortalized podocytes exposed to plasma from patients already treated with NS by using a microarray method (Panigrahi et al., 2021). There were four steroid‐sensitive NS serum‐induced profiles that clustered separately from the profiles induced by two steroid‐resistant NS samples (which clustered along with two healthy controls). Additionally, the differentially expressed genes did not overlap with our list of differentially expressed genes. In contrast to several significantly regulated pathways in our study, Panigrahi et al. found no pathways, as they admittedly limited the ability to detect any pathways due to the low sample count and the long‐known lower dynamic range of the microarray method compared to that of RNA sequencing (Zhao et al., 2014).

Several serum biomarkers have been found to be associated with one of the two major histological subtypes of NS, MCD or FSGS (Candelier & Lorenzo, 2020). However, as the original assumption that MCD and FSGS correspond to steroid‐sensitive and steroid‐resistant NS patients, respectively (Cameron, 1968; Gault & Muehrcke, 1983; Schulman et al., 1988), has been refuted (Narla & Swiatecka‐Urban, 2020; Trautmann et al., 2015; Vivarelli et al., 2017), these biomarkers (as well as biopsy results) are not useful in predicting the glucocorticoid treatment response. More promising markers for the prediction of glucocorticoid treatment response in NS patients might be linked to the immune system, particularly cytokines (Candelier & Lorenzo, 2020). In our study, we observed a decrease in the expression of the *IL18* gene, which encodes a proinflammatory marker (interleukin 18), in the steroid‐sensitive NS group compared to the steroid‐resistant NS group. Interleukin 18 induces the expression of the cytokines IL1, IL4, IL13, and IFN‐γ and activates both innate and acquired immune responses (Ihim et al., 2022; Rex et al., 2020). Patients with steroid‐sensitive NS have higher serum interleukin 18 concentrations at manifestation or relapse than at disease remission (Kiliś‐Pstrusińska et al., 2008; Printza et al., 2008). Interleukin 18 is an activator of the NLRP3 inflammasome and was experimentally verified to be involved in the pathogenesis of podocyte injury and proteinuria development (Fu et al., 2017). However, how these observations translate to our finding of downregulated *IL18* in serum‐exposed podocytes is unclear, as is the potential link between serum‐mediated immune processes and the podocyte response. Interestingly, the association between the immune system and NS was also described in a cytokine profiling study in which paired plasma samples collected before and 6–10 weeks after initial glucocorticoid treatment were examined in 26 steroid‐sensitive and 14 steroid‐resistant NS patients. A study showed that, compared with steroid‐resistant NS patients, steroid‐sensitive NS patients have a decreased concentration of IL7 and increased concentrations of IL9 and MCP1 (Agrawal et al., 2021). The same research group also performed whole proteomic profiling of plasma samples and identified 13 additional proteins mostly connected to extracellular matrix organization, posttranslational modification of proteins, metabolism of lipids and lipoproteins, cellular transport, and hemostasis (Agrawal et al., 2020). The 13 proteins combined with the three cytokines were able to distinguish patients according to their response to glucocorticoid treatment. These results highlight that NS prognosis may be predicted based on a parallel assessment of multiple molecular factors rather than relying on a single biomarker. To pursue such an approach, massive parallel assessment methods such as proteomics or transcriptomics are needed and ideally will be implemented together in a particular study.

The strengths of our study include the careful collection of treatment‐naive sera from NS patients who later developed steroid sensitivity. The standard collection procedure was followed for all serum samples, and there were no differences in serum storage. Furthermore, using technical replicates, we were able to demonstrate that the within‐serum variability in the transcriptome response was low and that it was clearly much lower than the difference between steroid‐sensitive and steroid‐resistant NS patient sera (Figure 1). Among the limitations, we were not able to collect a sufficient number of steroid‐resistant NS samples directly at disease manifestation. This is due to the rarity of steroid‐resistant NS. After recovering sera, collected later during the disease course, from steroid‐resistant NS patients, we carefully recruited only those subjects who had long‐term remission and had been in remission for at least 6 months without any immunosuppressive drugs at the time of sampling. Cyclosporine A‐induced T‐cell function suppression (measured by the expression of IL2 and IFN‐γ) was found to be fully restored after 4 days of drug withdrawal in dogs (Narayanan et al., 2020). In addition, extracellular matrix protein expression in trabecular meshwork tissue was normalized after 4 weeks of dexamethasone cessation (Li et al., 2017). Therefore, a 6‐month washout period represents the maximum amount of care taken to reduce the risk of bias caused by immunosuppressive treatment. In addition, the clustering of steroid‐sensitive NS outlier (i.e., sample free from any treatment) with steroid‐resistant NS samples suggested that treatment‐related bias is very improbable. With this collection strategy, we believe that we have avoided the possible influence of glucocorticoids or other immunosuppressive therapies that could affect the transcriptomic data and cover the real effect of patient sera in both groups. Another limitation may be the use of a model of human immortalized podocytes. Despite being verified and widely used, we cannot anticipate that the transcriptomic findings in immortalized podocytes resemble the situation in natural living podocytes of the kidneys in our patients. Our study was also unable to explore differences between the sexes.

In conclusion, to the best of our knowledge, this is the first study to demonstrate that, compared with sera from children with steroid‐resistant NS, sera from treatment‐naive children with steroid‐sensitive NS induce significantly different transcriptome profiles of in vitro cultured immortalized human podocytes. The steroid‐sensitive NS serum induces stress in podocytes, which likely mitigates the negative effects of the NS by enhancing DNA repair, cellular proliferation, and protein translation. However, further exploration of a larger cohort is needed to verify whether clinically distinct types of NS may be differentiated by their specific transcriptomic profiles and whether this information may help to reveal the pathophysiology of the steroid response.

## AUTHOR CONTRIBUTIONS

MB performed cultivation of cells, isolation of RNA, preparation of RNA libraries, and drafted the first manuscript version. OC performed RNA‐Seq data processing to get gene expression values and differential gene expression analysis. VS performed gene set enrichment analysis. KP helped with the wet‐lab work. ES collected the patients' samples and clinical data. JZ collected the patients' samples and clinical data. MS provided the human immortalized podocyte cell line. OS designed the study, supervised the wet lab procedures, and drafted the manuscript. All authors have revised and approved the final version of the manuscript.

## FUNDING INFORMATION

This study was supported by Charles University, project GA UK No. 384119, and by the Project for the Conceptual Development of Research Organization, Motol University Hospital (Ministry of Health, Czech Republic; 00064203) and the Research and Development for Innovation Operational Programme (Ministry of Education, Youth and Sports, Czech Republic, co‐financed by the EU; CZ.1.05/4.1.00/16.0337).

## CONFLICT OF INTEREST STATEMENT

The authors declare no competing interests.

## Supporting information


Data S1.

## Data Availability

The datasets generated and analyzed during the current study are available in the NCBI Gene Expression Omnibus (GEO) repository (accession code: GSE215231).
