# Supplementary material for: Nephrotic syndrome sera induce different transcriptomes in podocytes based on the steroid response
Source: Physiol Rep. 2024 Feb 2;12(3):e15932. doi: 10.14814/phy2.15932 (PMC10837055; doi:10.14814/phy2.15932)
Supplement: Supplementary file 1 — Data S1. [file PHY2-12-e15932-s001.pdf]

Nephrotic syndrome sera induce different transcriptomes in podocytes based on the steroid response

Supplementary material

Content:

Supplementary methods
Supplementary references

Supplementary methods: Gene set enrichment analysis

First, all genes were ordered in a ranked list L using a signal-to-noise ratio as defined in Maleki (1), 2.2.1. Univariate Functional Class Scoring Methods, i.e. gene g_j in a list L has a score r_j. Derivation of the Enrichment score is based on Sumbramanian (2). Let G denote the analyzed pathway and let

$$P_{hit}(G,i) = \sum_{\substack{g_j \in G \\ j \le i}} \frac{|r_j|}{K_G},$$

where K_G is a normalizing constant given by a sum of absolute values of r_j in the pathway G, i.e.

$$K_G = \sum_{g_j \in G} |r_j|.$$

Analogously, we define

$$P_{miss}(G,i) = \sum_{\substack{g_j \notin G \\ j \le i}} \frac{1}{N - N_G},$$

where N is a total number of genes in the list L and N_G is a number of genes in the pathway G.

Then we calculate the running Enrichment score for the pathway G as follows:

$$RES_k(G) = \sum_{i \le k} [P_{hit}(G,i) - P_{miss}(G,i)], \qquad k = 1, 2, \ldots, N.$$

Now we determine the index m maximizing deviation of the running Enrichment score from 0:

$$m = \arg \max_{k=1,2,\ldots,N} |RES_k(G)|.$$

Finally, the Enrichment score ES for the pathway G is calculated as

$$ES(G) = RES_m(G),$$

which attains either positive or negative value, as a consequence of the pathway up-regulation or down-regulation, respectively.

Supplementary references

1. **Maleki F, Ovens K, Hogan DJ, and Kusalik AJ**. Gene Set Analysis: Challenges, Opportunities, and Future Research. *Front Genet* 11: 654, 2020.
2. **Subramanian A, Tamayo P, Mootha VK, Mukherjee S, Ebert BL, Gillette MA, Paulovich A, Pomeroy SL, Golub TR, Lander ES, and Mesirov JP**. Gene set enrichment analysis: a knowledge-based approach for interpreting genome-wide expression profiles. *Proc Natl Acad Sci U S A* 102: 15545-15550, 2005.
